# Temporal trends and disparities in mesenteric ischemia–associated cardiovascular mortality among U.S. adults aged ≥45 years, 1999–2023

**DOI:** 10.3389/fcvm.2026.1920942

**Published:** 2026-10-09

**Authors:** Muhammad Atif Mazhar, Taha Alam, Yusra Fazli, Dhvanit Rajdeep, Bhaskar Kambhampati, Usama Tufail, Muhammad Mukhlis, Sohaima Kamal, Noor Un Nisa, Rafiullah Hotak, Sadia Qazi

**Affiliations:** 1 Department of Anatomy, College of Medicine, Alfaisal University, Riyadh, Saudi Arabia; 2 Department of Medicine, Dow University of Health Sciences, Karachi, Pakistan; 3 Department of Medicine, Ziauddin University, Karachi, Pakistan; 4 Department of Medicine, Caucasus International University, Tbilisi, Georgia; 5 Department of Medicine, Rangaraya Medical College, Andhra Pradesh, India; 6 Department of Medicine, King Edward Medical University, Lahore, Pakistan; 7 Department of Medicine, Ayub Medical College, Abbottabad, Pakistan; 8 Department of Medicine, Jinnah Sindh Medical University, Karachi, Pakistan; 9 Nangarhar University Faculty of Medicine, Jalalabad, Afghanistan

**Keywords:** cardiovascular diseases, epidemiology, health disparities, joinpoint regression, mesenteric ischemia, mortality, population-based study 4, United States

## Abstract

**Background:**

Mesenteric ischemia (MI) is a highly fatal vascular condition associated with cardiovascular disease, but long-term national mortality patterns remain poorly described.

**Objective:**

To evaluate national mortality trends, demographic heterogeneity, and geographic variations in MI and cardiovascular mortality among U.S. adults aged ≥45 years from 1999 to 2023.

**Methods:**

We conducted a population-based analysis using CDC WONDER Multiple Cause of Death data. AAMRs per 100,000 population were computed, and temporal trends were evaluated using Joinpoint regression to estimate annual percent changes (APC) and average annual percent change (AAPC) with 95% confidence intervals. The analyses were stratified by sex, race/ethnicity, age group, urbanization, and census region.

**Results:**

From 1999 to 2023, 61,543 deaths involving MI and cardiovascular diseases were recorded. The overall AAMR declined from 4.68 in 1999 to 1.47 in 2023, with an AAPC of −4.73% (95% CI: −5.16 to −4.30). Mortality decreased significantly until 2015 (APC: −6.86), followed by an increase thereafter (APC: +1.25). Women consistently had higher mortality than men and experienced a significant post-2016 increase, whereas men's rates plateaued. Non-Hispanic (NH) Black adults had the highest early rates (5.21 in 1999) and experienced a significant rise after 2016 (APC: 2.87%). Adults aged 45–64 years experienced the sharpest reversal, with mortality rising after 2013. Regionally, the South showed the greatest late increase, and urban–rural disparities persisted. Most deaths occurred in inpatient medical facilities (85.65%).

**Conclusion:**

After nearly two decades of decline, MI-associated cardiovascular mortality has rebounded in the U.S. since 2015, underscoring the need for renewed prevention and equity-focused care.

## 1 Introduction

Mesenteric ischemia is one of the lethal manifestations of vascular diseases. It consists of inadequate intestinal perfusion which eventually leads to bowel infarction, sepsis and death. Acute and chronic disorders of the intestine are classified under the International Classification of Diseases Tenth Revision (ICD-10) codes K55.0 and K55.1. These disorders are associated with high mortality especially when there is delay in diagnosis and revascularization (1, 2). Although diagnostic imaging and revascularization techniques have advanced, mortality remains high. This highlights the importance of early recognition and timely intervention.

Cardiovascular disease (CVD) which falls under ICD-10 codes I00-I99 play a central role in the pathophysiology of mesenteric ischemia. For example, conditions like diffuse atherosclerosis and atrial fibrillation may lead to mesenteric arterial occlusion. Whereas, conditions like heart failure and systemic hypoperfusion may contribute to non-occlusive mesenteric ischemia (2, 3). Mesenteric ischemia frequently occurs in individuals with pre-existing cardiovascular disease. This further highlights their overlapping vascular risk factors and mechanisms of injury. Clinical guidelines also emphasize that mesenteric ischemia events rarely arise in isolation and are rather seen as co-existing with advanced cardiovascular pathology (4). Over the past two decades, improvements in cardiovascular prevention, medical treatments and revascularization have improved cardiovascular mortality trends in the US (5). As a consequence, this has also led to an increase in the aging population as patients with coronary and cerebrovascular disease survive longer and hence may also contribute to the growing burden of chronic vascular comorbidity. However, recent national data also shows that the improvements in cardiovascular mortality have slowed in the mid-2010s and reversed during and after the COVID-19 pandemic (6). The impact of these shifts downstream to vascular complications like mesenteric ischemia remain incompletely understood.

Previous studies of mesenteric ischemia have largely been case series, cohorts, or short-term outcome studies. This limited their ability to assess population level mortality patterns (1, 7). Given the rarity of mesenteric ischemia, national mortality databases offer a critical opportunity to examine trends that cannot be captured by single center studies. These include temporal patterns, demographic disparities and geographic variations. A recent CDC WONDER-based analysis examined mortality trends in vascular intestinal disorders broadly, including mesenteric ischemia, ischemic colitis, and intestinal angiodysplasia from 1999 to 2020, and demonstrated substantial demographic heterogeneity in mortality (7). However, this analysis did not isolate mesenteric ischemia specifically, did not evaluate its relationship with cardiovascular comorbidity, and predated the COVID-19 pandemic, a period associated with a documented reversal in cardiovascular mortality trends nationally. Given that mesenteric ischemia and cardiovascular disease share overlapping vascular pathophysiology and risk factors, and that CVD-related deaths have shown divergent trends in the past decade, it remains unknown whether mortality attributable to mesenteric ischemia with co-existing cardiovascular disease has followed the same trajectory, plateaued, or worsened and whether this varies across demographic and geographic strata. To our knowledge, no prior study has used a national multiple-cause-of-death framework to specifically examine mesenteric ischemia mortality in the context of cardiovascular comorbidity across this extended and updated time frame.

Understanding mortality trends in mesenteric ischemia is relevant in relation to its cardiovascular risk factor control and access to specialized vascular care. National cardiovascular surveillance data demonstrate marked differences in disease burden by age, race and ethnicity and geography. It also highlights that underserved populations, particularly those in resource-limited settings, experience disproportionate adverse outcomes (5, 8). Mesenteric ischemia management is very time-sensitive hence delays in diagnosis, interfacility transfer and access to specialized care may further exacerbate these disparities.

To address these gaps, we conducted a nationwide, population-based analysis using the Centers for Disease Control and Prevention Wide-Ranging Online Data for Epidemiologic Research (CDC WONDER) Multiple Cause of Death database. We evaluated long-term mortality trends associated with acute and chronic mesenteric ischemia (ICD-10 K55.0-K55.1) with cardiovascular disease (ICD-10 I00-I99) among U.S. adults aged 45 years and older from 1999 to 2023. We aimed to analyse temporal trends in age-adjusted mortality, assess disparities across demographic and geographic strata and identify populations at increased risk.

## 2 Methods

### 2.1 Study setting and population

This nationwide retrospective population-based study utilized mortality data retrieved from the Centers for Disease Control and Prevention (CDC) Wide-Ranging Online Data for Epidemiologic Research (CDC WONDER) database. We evaluated temporal trends in mortality associated with Mesenteric Ischemia (MI) and Cardiovascular Disease (CVD) among U.S. adults aged ≥45 years from 1999 to 2023. Mortality data were obtained from the Multiple Cause-of-Death (MCD) Public-Use files, which include certified death records from all 50 U.S. states and the District of Columbia.

Deaths related to mesenteric ischemia were identified using ICD-10 codes K55.0 (acute vascular disorders of intestine) and K55.1 (chronic vascular disorders of intestine) (7). These codes were analyzed as a combined mesenteric-ischemia group in the present study. Cardiovascular disease was identified using ICD-10 codes I00–I99 (9). For the multiple-cause-of-death definition, both the mesenteric ischemia and cardiovascular disease code groups were considered, with the relevant codes eligible to appear as either the underlying or a contributing cause of death.

As this study exclusively utilized publicly available, de-identified data, institutional review board approval was not required. The study adhered to the Strengthening the Reporting of Observational Studies in Epidemiology (STROBE) guidelines to ensure methodological transparency and reporting quality (10).

### 2.2 Data extraction

Data extraction was independently conducted by one author and verified by two additional reviewers to ensure accuracy and consistency. Extracted variables included year of death, age group, sex, race/ethnicity, and urban–rural classification.

Age was categorized into middle-aged adults (45–64 years) and older adults (≥65 years). Race/ethnicity was categorized as: Non-Hispanic (NH) White, NH Black/African American, NH Asian or Pacific Islander, NH American Indian/Alaska Native, and Hispanic/Latino. Urban–rural stratification was performed using the National Center for Health Statistics Urban–Rural Classification Scheme, and subsequently grouped into “Metro” (large central, large fringe, medium, and small metropolitan areas) and “Non-Metro” (micropolitan and rural non-core areas) (11). Urban-rural data were available only through 2020; therefore, analyses involving these variables were restricted accordingly.

### 2.3 Statistical analysis

Crude Mortality Rates (CMR) and Age-Adjusted Mortality Rates (AAMR) were calculated per 100,000 population. Age adjustment was performed using the 2,000 U.S. Standard Population to account for temporal demographic shifts (12). Rates derived from <20 deaths were considered statistically unreliable and were suppressed.

Temporal mortality trends were analyzed using the Joinpoint Regression Program, version 5.4.0.0 (National Cancer Institute) (13). Annual age-adjusted mortality rates and their corresponding standard errors were entered into Joinpoint, with the supplied standard errors used as the uncertainty estimates for the annual rates. The standard grid-search Joinpoint method was used, allowing 0 to 4 joinpoints, with a minimum of two observations required before the first joinpoint, after the final joinpoint, and between adjacent joinpoints. Model selection was performed using the permutation test with an overall significance level of 0.05 and 4,499 permutations. This approach was selected to identify data-supported changes in temporal mortality trends without imposing prespecified calendar breakpoints. Annual Percentage Change (APC) was estimated for each fitted segment, and Average Annual Percentage Change (AAPC) was estimated over the specified study interval. Ninety-five percent confidence intervals for APC and AAPC were calculated using the parametric confidence-interval method implemented in Joinpoint. Confidence intervals accompanying the annual mortality-rate estimates were obtained with the rate estimates from CDC WONDER. Statistical significance was assessed at *p* < 0.05. To assess robustness to the COVID-19 period, the Joinpoint analyses were repeated after restricting the study interval to 1999–2019.

## 3 Results

### 3.1 Annual trends for MI and CVD mortality

A total of 61,543 deaths were recorded with mesenteric ischemia and CVD among individuals in the United States between 1999 and 2023. The AAMR for mesenteric ischemia and CVD-related deaths was 4.68 in 1999 and 1.47 in 2023. While the rate has generally declined throughout the study period, the pace of the decrease has varied. The overall AAMR for deaths related to mesenteric ischemia and CVD showed a strong initial decline, with an APC of −9.17 (95% CI: −10.42 to −7.91) from 1999 to 2004, which then decelerated slightly to −6.86 (95% CI: −7.40 to −6.31) for the 2004 to 2015 period. This was followed by a shift in the trend in the most recent period, 2015 to 2023, indicating a slight increase with an APC of 1.25 (95% CI: 0.38 to 2.13). The 95% confidence interval indicates that, although the reversal is statistically significant, the annual pace of increase plausibly ranges from a modest 0.4% to a more pronounced 2.1% per year, corresponding to a rise in AAMR from a nadir of 1.32 in 2015 to 1.47 in 2023, an absolute increase of 0.15 per 100,000 (an 11.4% relative rise from the 2015 low). Because the present analysis used the combined ICD-10 K55.0–K55.1 code group, separate mortality trends for acute and chronic mesenteric ischemia were not estimated (Figure 1; Table 1 and Supplementary Table S1).

**Figure 1:**
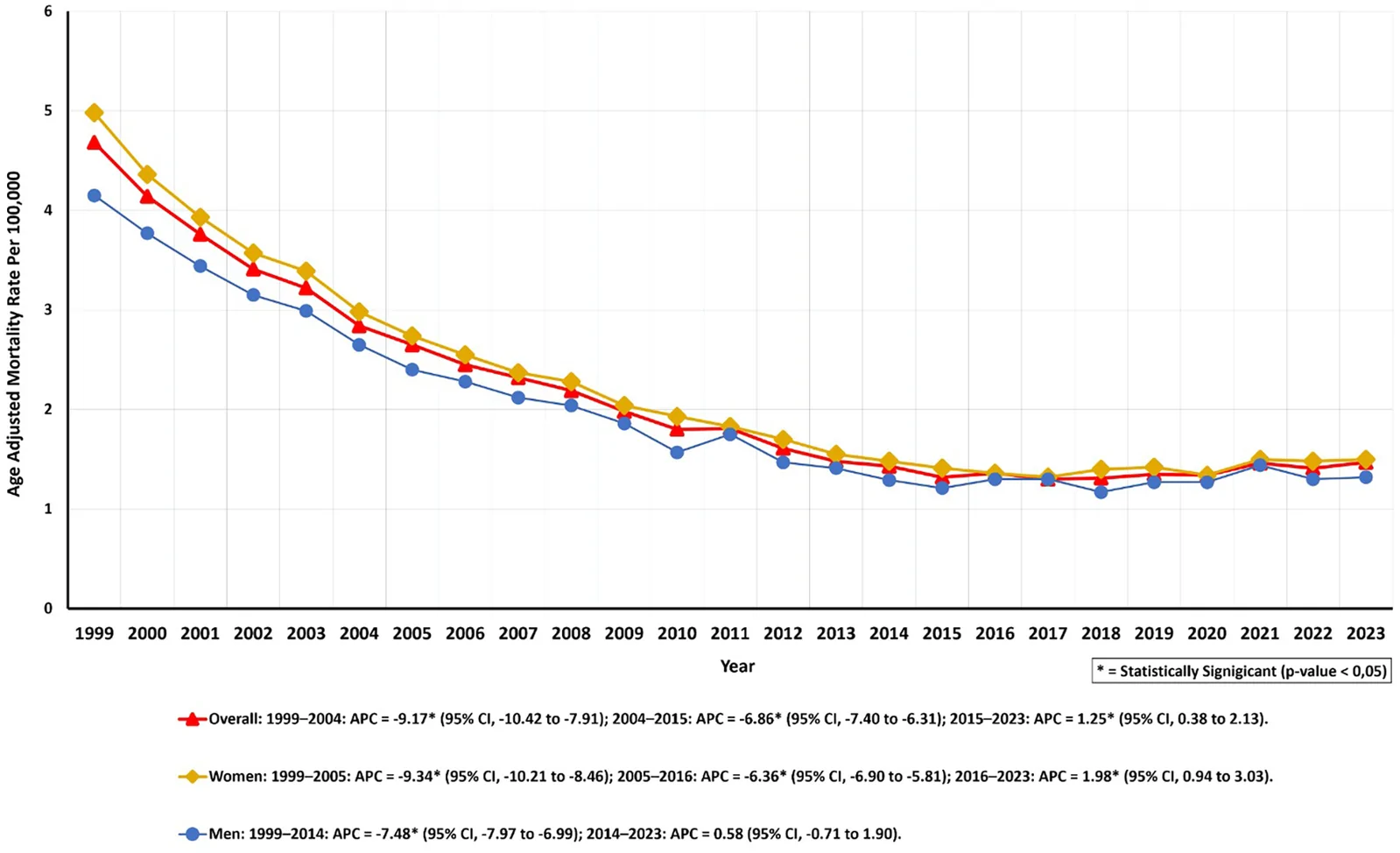
Overall and sex stratified age-adjusted mortality rates (per 100,000) for mesenteric ischemia and cardiovascular disease related mortality among U.S. adults aged ≥45, 1999 to 2023.

**Table 1:** Annual percent change (APC) and average annual percent change (AAPC) of mesenteric ischemia and cardiovascular disease, shown as age-adjusted mortality rates per 100,000 among adults aged ≥45 in the United States, 1999–2023.

Year	APC (95% CI)	*p*-value for APC	AAPC (95% CI)	*p*-value for AAPC
Overall				
1999 to 2004	−9.17 (−10.42 to −7.91)	< 0.000001		
2004 to 2015	−6.86 (−7.40 to −6.31)	< 0.000001		
2015 to 2023	1.25 (0.38 to 2.13)	0.007401		
1999–2023			−4.73 (−5.16 to −4.30)	< 0.000001
Sex				
Women				
1999 to 2005	−9.34 (−10.21 to −8.46)	< 0.000001		
2005 to 2016	−6.36 (−6.90 to −5.81)	< 0.000001		
2016 to 2023	1.98 (0.94 to 3.03)	0.000843		
1999–2023			−4.77 (−5.18 to −4.36)	< 0.000001
Men				
1999 to 2014	−7.48 (−7.97 to −6.99)	< 0.000001		
2014 to 2023	0.58 (−0.71 to 1.90)	0.361281		
1999–2023			−4.53 (−5.06 to −4.00)	< 0.000001
Race				
NH Asian or Pacific Islander				
1999 to 2014	−8.37 (−9.86 to −6.85)	< 0.000001		
2014 to 2023	−0.74 (−4.07 to 2.71)	0.656239		
1999–2023			−5.58 (−7.02 to −4.11)	< 0.000001
NH Black or African American				
1999 to 2006	−10.18 (−12.03 to −8.30)	< 0.000001		
2006 to 2016	−6.08 (−7.80 to −4.33)	0.000002		
2016 to 2023	2.87 (0.26 to 5.56)	0.032905		
1999–2023			−4.80 (−5.89 to −3.70)	< 0.000001
NH White				
1999 to 2005	−8.45 (−9.45 to −7.43)	< 0.000001		
2005 to 2015	−6.65 (−7.35 to −5.95)	< 0.000001		
2015 to 2023	1.38 (0.31 to 2.46)	0.014619		
1999–2023			−4.51 (−5.00 to −4.03)	< 0.000001
Hispanic or Latino				
1999 to 2015	−7.89 (−8.95 to −6.81)	< 0.000001		
2015 to 2023	2.96 (−0.19 to 6.21)	0.064283		
1999–2023			−4.40 (−5.56 to −3.23)	< 0.000001
Age group				
45–64 Years				
1999 to 2013	−6.64 (−7.63 to −5.65)	< 0.000001		
2013 to 2023	3.24 (1.39 to 5.13)	0.001513		
1999–2023			−2.65 (−3.53 to −1.75)	< 0.000001
65–85+ Years				
1999 to 2004	−9.16 (−10.56 to −7.74)	< 0.000001		
2004 to 2015	−7.18 (−7.84 to −6.52)	< 0.000001		
2015 to 2023	0.47 (−0.63 to 1.58)	0.383479		
1999–2023			−5.13 (−5.64 to −4.61)	< 0.000001
Urbanization				
Metropolitan				
1999 to 2004	−9.61 (−10.89 to −8.31)	< 0.000001		
2004 to 2015	−6.81 (−7.44 to −6.17)	< 0.000001		
2015 to 2020	−0.51 (−2.36 to 1.37)	0.565902		
1999–2020			−6.03 (−6.60 to −5.46)	< 0.000001
Non-metropolitan				
1999 to 2015	−6.51 (−6.96 to −6.07)	< 0.000001		
2015 to 2020	1.92 (−1.55 to 5.51)	0.263092		
1999–2020			−4.57 (−5.37 to −3.77)	< 0.000001
Census Region				
Northeast				
1999 to 2014	−7.70 (−8.34 to −7.06)	< 0.000001	2001–2006	
2014 to 2023	0.26 (−1.68 to 2.23)	0.787439	2006–2015	
1999–2023			−4.79 (−5.55 to −4.03)	< 0.000001
Midwest				
1999 to 2015	−6.99 (−7.50 to −6.47)	< 0.000001		
2015 to 2023	1.47 (−0.46 to 3.44)	0.12896		
1999–2023			−4.25 (−4.91 to −3.58)	< 0.000001
South				
1999 to 2006	−9.79 (−11.11 to −8.46)	< 0.000001		
2006 to 2017	−6.18 (−7.24 to −5.10)	< 0.000001		
2017 to 2023	3.16 (0.51 to 5.87)	0.021709		
1999–2023			−5.02 (−5.84 to −4.19)	< 0.000001
West				
1999 to 2014	−7.33 (−7.95 to −6.71)	< 0.000001		
2014 to 2023	1.09 (−0.51 to 2.71)	0.173108		
1999–2023			−4.26 (−4.92 to −3.60)	< 0.000001

### 3.2 MI and CVD mortality stratified by sex

The AAMR for both males and females followed a similar overall trend but with different magnitudes. Throughout the entire study period, females consistently maintained a higher age-adjusted mortality rate (AAMR) than males (overall AAMR females: 2.26; 95% CI: 2.14 to 2.37; males: 2.00; 95% CI: 1.87 to 2.12). In 1999, the AAMR for females was 4.98 (95% CI: 4.79 to 5.16), which decreased to 1.50 (95% CI: 1.42 to 1.58) in 2023. Overall, the AAMR in females showed a substantial decrease from 1999 to 2023 with an AAPC of −4.77 (95% CI: −5.18 to −4.36).

The trend for females began with a sharp decline from 1999 to 2005 (APC: −9.34; 95% CI: −10.21 to −8.46), followed by a continued but slower decrease between 2005 and 2016 (APC: −6.36; 95% CI: −6.90 to −5.81), and finally shifting to an increase from 2016 to 2023 (APC: 1.98; 95% CI: 0.94 to 3.03). This corresponds to a rise in AAMR from 1.36 in 2016 to 1.50 in 2023 (+0.14 per 100,000; a 10.3% relative increase); the confidence interval indicates the annual increase is unlikely to be trivial, plausibly ranging from just under 1% to just over 3% per year.

Similarly, the AAMR for males was 4.15 (95% CI: 3.94 to 4.36) in 1999, decreasing to 1.32 (95% CI: 1.23 to 1.41) in 2023. This significant decrease was reflected in an AAPC of −4.53 (95% CI: −5.06 to −4.00). The rate for males showed a consistent decline from 1999 to 2014 (APC: −7.48; 95% CI: −7.97 to −6.99), followed by a period of stability from 2014 to 2023 (APC: 0.58; 95% CI: −0.71 to 1.90). As the 95% CI spans zero, this estimate is equally consistent with a small ongoing decline or a modest increase, and is best read as a plateau rather than a directional trend (Figure 1; Table 1 and Supplementary Table S1).

### 3.3 MI and CVD mortality stratified by race

The AAMRs for mesenteric ischemia and CVD-related deaths showed an overall downward trend across all racial groups from 1999 to 2023, though the magnitude and recent reversals varied substantially. The highest AAMRs were observed among American Indian or Alaska Native individuals (mean overall AAMR: 3.56; 95% CI: 1.78 to 4.95), followed by Black or African American (mean overall AAMR: 2.24; 95% CI: 1.95 to 2.53), White (mean overall AAMR: 2.20; 95% CI: 2.11 to 2.29), Hispanic or Latino (mean overall AAMR: 2.00; 95% CI: 1.68 to 2.33), and finally Asian or Pacific Islander (mean overall AAMR: 1.11; 95% CI: 0.80 to 1.51).

The AAMR for American Indian or Alaska Native adults was unreliable in 1999, recorded at 5.75 (95% CI: 3.69 to 8.56) in 2000, and decreased to 2.18 (95% CI: 1.32 to 3.41) in 2023. Due to small numbers, a detailed trend analysis could not be performed for this cohort.

Asian or Pacific Islanders experienced a decrease in AAMR from 2.62 (95% CI: 1.95 to 3.46) in 1999 to 0.67 (95% CI: 0.51 to 0.88) in 2023. The rate showed a rapid decrease from 1999 to 2014 (APC: −8.37; 95% CI: −9.86 to −6.85), followed by a period of statistical stability from 2014 to 2023 (APC: −0.74; 95% CI: −4.07 to 2.71; *p*-value=0.66).

For Black or African American individuals, the AAMR fell from 5.21 (95% CI: 4.71 to 5.72) in 1999 to 1.57 (95% CI: 1.36 to 1.77) in 2023. The AAMR initially decreased rapidly from 1999 to 2006 (APC: −10.18; 95% CI: −12.03 to −8.30), then continued to decline at a slower pace from 2006 to 2016 (APC: −6.08; 95% CI: −7.80 to −4.33). However, the trend reversed significantly, rising from 2016 to 2023 (APC: 2.87; 95% CI: 0.26 to 5.56; *p*-value=0.03). This reflects an absolute rise in AAMR from 1.17 in 2016 to 1.57 in 2023 (+0.40 per 100,000; a 34.2% relative increase), proportionally the largest late-period reversal of any racial/ethnic group in this analysis, although the wide interval (0.26 to 5.56) reflects the smaller number of deaths in this stratum and should temper firm conclusions about the precise annual pace.

White adults had an AAMR of 4.63 (95% CI: 4.48 to 4.78) in 1999, which declined to 1.51 (95% CI: 1.43 to 1.58) in 2023. The trend followed a similar pattern with a sharp initial drop from 1999 to 2005 (APC: −8.45; 95% CI: −9.45 to −7.43), followed by a slower decline between 2005 and 2015 (APC: −6.65; 95% CI: −7.35 to −5.95) and a significant increase from 2015 to 2023 (APC: 1.38; 95% CI: 0.31 to 2.46; *p*-value=0.01). This corresponds to a rise in AAMR from 1.36 in 2015 to 1.51 in 2023 (+0.15 per 100,000; an 11.0% relative increase).

The AAMR in the Hispanic population decreased from 4.86 (95% CI: 4.18 to 5.54) in 1999 to 1.39 (95% CI: 1.20 to 1.59) in 2023. The rate decreased rapidly from 1999 to 2015 (APC: −7.89; 95% CI: −8.95 to −6.81), then showed a non-significant increase from 2015 to 2023 (APC: 2.96; 95% CI: −0.19 to 6.21; *p*-value=0.06) (Figure 2; Table 1 and Supplementary Table S2).

**Figure 2:**
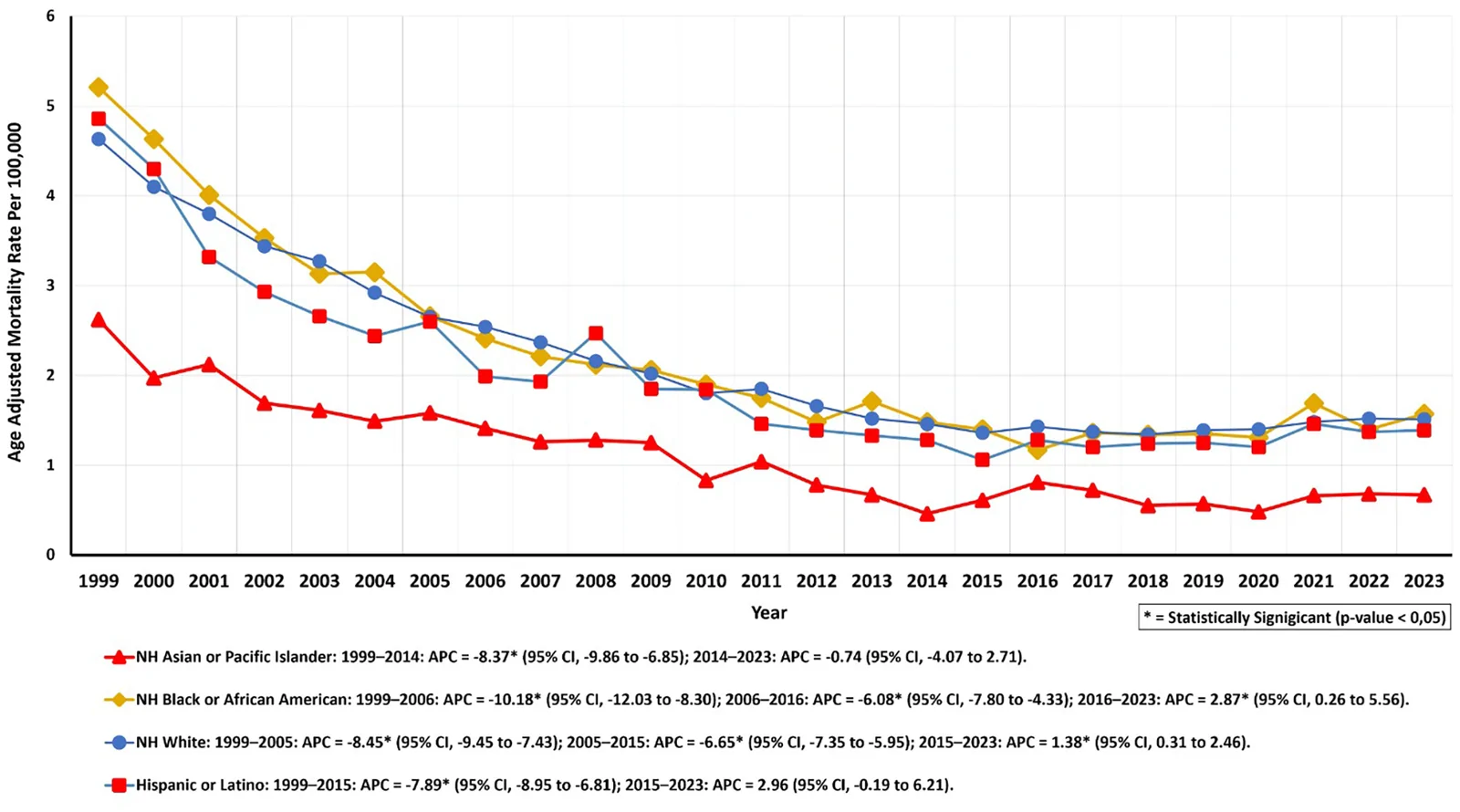
Race stratified age-adjusted mortality rates (per 100,000) for mesenteric ischemia and cardiovascular disease related mortality among U.S. adults aged ≥45, 1999 to 2023.

### 3.4 MI and CVD mortality stratified by age group

The age group analysis showed significant disparities. Older adults (65-85 + years) had the highest mortality rate, with CMR decreasing from 11.04 (95% CI: 10.69 to 11.39) in 1999 to 2.97 (95% CI: 2.84 to 3.11) in 2023, resulting in a mean overall CMR of 5.02 (95% CI: 4.81 to 5.23). The CMR for deaths related to mesenteric ischemia and CVD exhibited a rapid initial decrease from 1999 to 2004 (APC: −9.16; 95% CI: −10.56 to −7.74), which then continued to decline between 2004 and 2015 (APC: −7.18; 95% CI: −7.84 to −6.52). A stabilization occurred in the most recent period from 2015 to 2023 (APC: 0.47; 95% CI: −0.63 to 1.58; *p*-value=0.38).

Similarly, middle-aged adults (45-64 years) exhibited a decrease in CMR from 0.98 (95% CI: 0.91 to 1.06) in 1999 to 0.52 (95% CI: 0.47 to 0.57) in 2023. The mean overall CMR for this age group was 0.54 (95% CI: 0.49 to 0.60). The rates initially decreased from 1999 to 2013 (APC: −6.64; 95% CI: −7.63 to −5.65), followed by a notable increase between 2013 and 2023 with an APC of 3.24 (95% CI: 1.39 to 5.13; *p*-value=0.0015). This corresponds to a rise in CMR from 0.40 in 2013 to 0.52 in 2023 (+0.12 per 100,000; a 30.0% relative increase), proportionally the largest reversal observed across any age stratum, though the wide interval (1.39 to 5.13) again reflects the comparatively small absolute number of deaths in this younger cohort (Figure 3; Table 1, and Supplementary Table S3).

**Figure 3:**
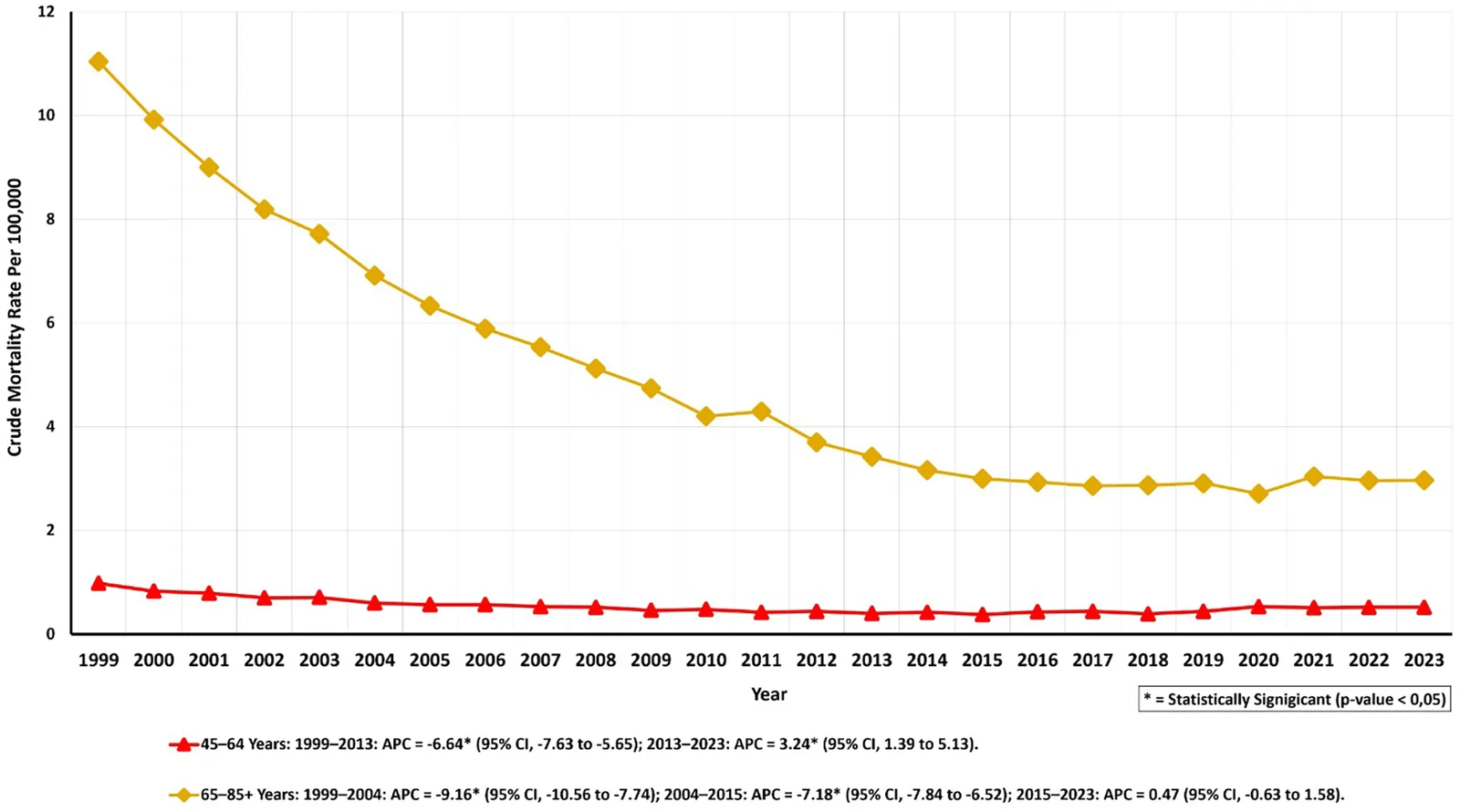
Age stratified crude mortality rates per 100,000 for mesenteric ischemia and cardiovascular disease related mortality in United States adults aged ≥45 years, 1999-2023.

### 3.5 MI and CVD mortality stratified by urbanization

By urbanization status, non-metropolitan areas (mean overall AAMR: 2.65; 95% CI: 2.43 to 2.88) had higher AAMRs than metropolitan areas (mean overall AAMR: 2.18; 95% CI: 2.09 to 2.27). The analysis showed a consistent downward trend in both regions. For metropolitan areas, the AAMR decreased from 4.56 (95% CI: 4.41 to 4.71) in 1999 to 1.21 (95% CI: 1.15 to 1.27) in 2020. The trend began with the sharpest decline from 1999 to 2004 (APC: −9.61; 95% CI: −10.89 to −8.31), followed by a slower decrease between 2004 and 2015 (APC: −6.81; 95% CI: −7.44 to −6.17), and then stabilized from 2015 to 2020 (APC: −0.51; 95% CI: −2.36 to 1.37; *p*-value=0.57).

For non-metropolitan regions, the AAMR was 5.03 (95% CI: 4.70 to 5.36) in 1999, decreasing significantly to 1.80 (95% CI: 1.63 to 1.97) by 2020. The trend notably reduced from 1999 to 2015 (APC: −6.51; 95% CI: −6.96 to −6.07), followed by a period of statistical stability between 2015 and 2020 (APC: 1.92; 95% CI: −1.55 to 5.51; *p*-value=0.26) (Figure 4; Table 1, Supplementary Table S4).

**Figure 4:**
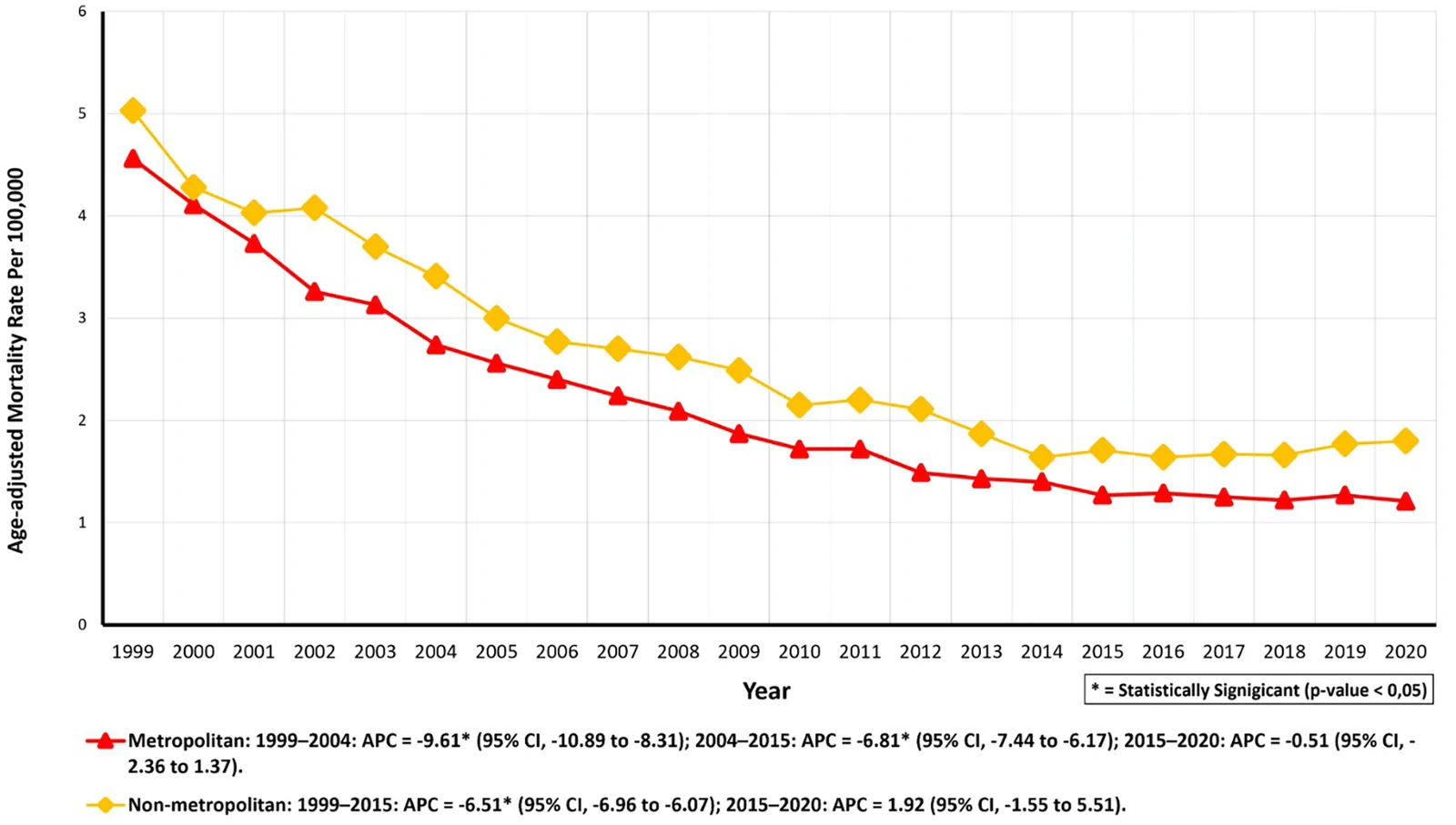
Urbanization stratified age-adjusted mortality rates per 100,000 for mesenteric ischemia and cardiovascular disease related mortality in United States adults aged ≥45 years, 1999-2020.

### 3.6 MI and CVD mortality stratified by census region

Over the course of the study, the highest mortality was observed in the Western region (mean AAMR: 2.35; 95% CI: 2.16 to 2.54), followed by the Midwestern (mean AAMR: 2.23; 95% CI: 2.05 to 2.40), Southern (mean AAMR: 2.15; 95% CI: 2.01 to 2.29), and Northeastern regions (mean AAMR: 1.90; 95% CI: 1.73 to 2.07).

The AAMR for the Western region was 4.67 (95% CI: 4.36 to 4.98) in 1999 and decreased to 1.64 (95% CI: 1.51 to 1.78) by 2023. The rate showed a significant decline from 1999 to 2014 (APC: −7.33; 95% CI: −7.95 to −6.71), followed by a period of stabilization from 2014 to 2023 (APC: 1.09; 95% CI: −0.51 to 2.71; *p*-value=0.17).

For the Midwest region, a decline in AAMR was seen from 4.58 (95% CI: 4.30 to 4.85) in 1999 to 1.50 (95% CI: 1.36 to 1.63) in 2023. The AAMR decreased significantly from 1999 to 2015 (APC: −6.99; 95% CI: −7.50 to −6.47), before stabilizing from 2015 to 2023 (APC: 1.47; 95% CI: −0.46 to 3.44; *p*-value=0.13).

Similarly, the AAMR for the Southern region decreased from 4.95 (95% CI: 4.71 to 5.18) in 1999 to 1.36 (95% CI: 1.26 to 1.45) in 2023. The AAMR fell rapidly from 1999 to 2006 (APC: −9.79; 95% CI: −11.11 to −8.46), followed by a continued but slower decline between 2006 and 2017 (APC: −6.18; 95% CI: −7.24 to −5.10). However, the trend reversed, with a significant rise from 2017 to 2023 (APC: 3.16; 95% CI: 0.51 to 5.87; *p*-value=0.02). This equates to an absolute rise in AAMR from 1.20 in 2017 to 1.36 in 2023 (+0.16 per 100,000; a 13.3% relative increase).

The AAMR for the Northeast was 4.25 (95% CI: 3.97 to 4.54) in 1999, and decreased to 1.30 (95% CI: 1.16 to 1.43) in 2023. The rate showed a sharp initial decrease from 1999 to 2014 (APC: −7.70; 95% CI: −8.34 to −7.06), followed by a period of stabilization from 2014 to 2023 (APC: 0.26; 95% CI: −1.68 to 2.23; *p*-value=0.79) (Figure 5; Table 1, Supplementary Table S5).

**Figure 5:**
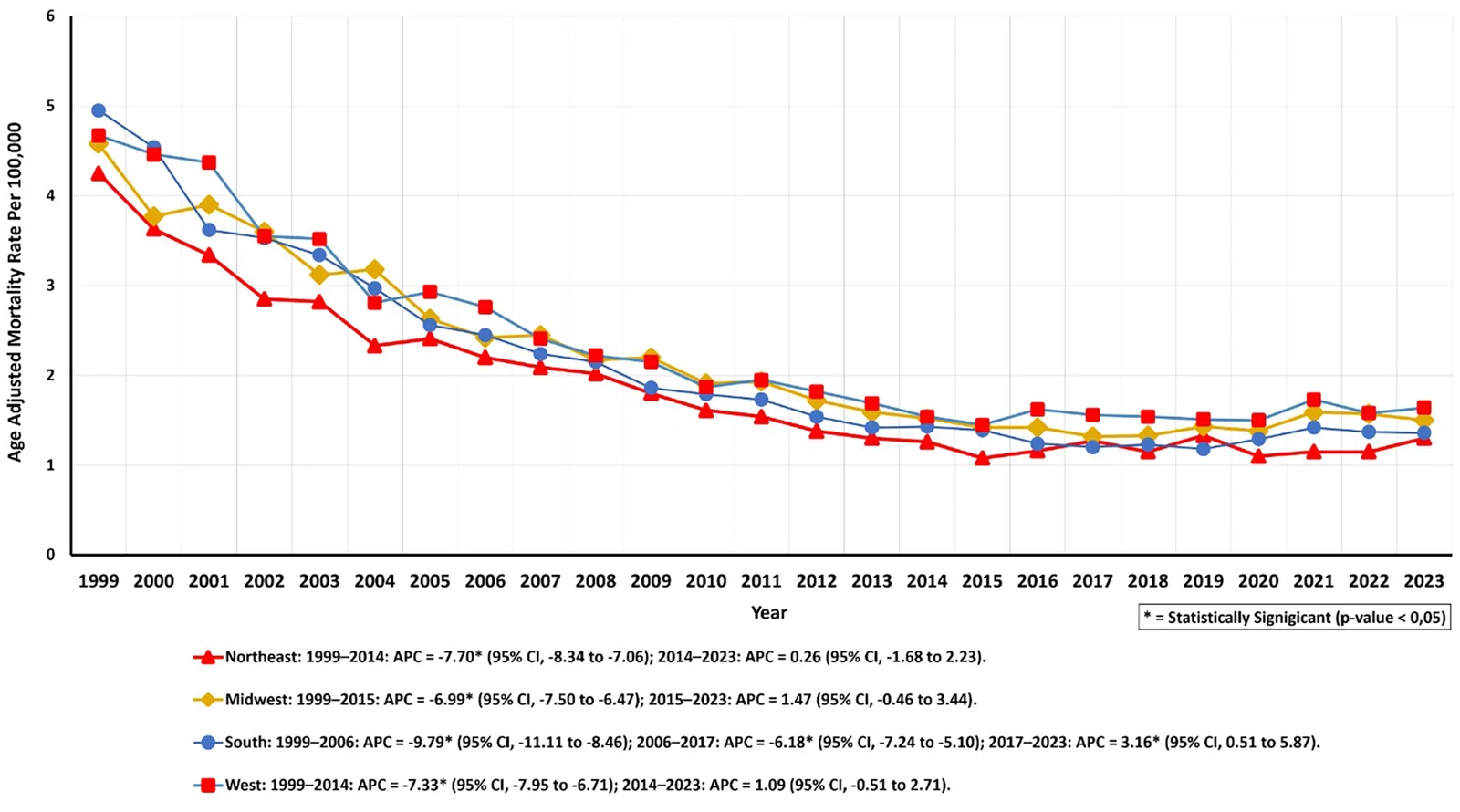
Census region stratified age-adjusted mortality rates per 100,000 for mesenteric ischemia and cardiovascular disease related mortality in United States adults aged ≥45 years, 1999-2023.

### 3.7 MI and CVD mortality stratified by states

Death rates revealed significant disparities across various states. From 1999 to 2020, the AAMR ranged from 3.24 (95% CI: 2.78 to 3.70) in Vermont to 1.53 (95% CI: 1.31 to 1.74) in Hawaii. The states that fell into the top 90th percentile are Vermont (3.24), Alaska (2.99), Idaho (2.89), Oklahoma (2.87), Wyoming (2.82), and Tennessee (2.73). The states that fell into the bottom 10th percentile are Hawaii (1.53), New Jersey (1.57), Arizona (1.59), Florida (1.61), Virginia (1.62), and Massachusetts (1.65).

Similarly, from 2021 to 2023, the AAMR ranged from 2.51 (95% CI: 2.10 to 2.92) in Oregon to 0.82 (95% CI: 0.59 to 1.10) in Connecticut. The states that fell into the top 90th percentile from 2021 to 2023 are Oregon (2.51), South Dakota (2.44), Washington (2.39), Minnesota (2.37), and Vermont (2.28), while those in the bottom 10th percentile are Connecticut (0.82), Hawaii (0.83), New Jersey (0.92), Illinois (1.01), and Louisiana (1.03) (Supplementary Figures S1–S3 and Supplementary Table S6).

### 3.8 MI and CVD mortality stratified by place of death

Information on the location of death was available for 61,534 decedents. The overwhelming majority of deaths occurred within an inpatient medical facility (*n* = 52,702; 85.65%). When all medical-facility settings are combined, inpatient, outpatient/emergency department, dead-on-arrival, and status-unknown, this proportion rises to 88.97% (*n* = 54,744). Deaths outside a medical facility were comparatively uncommon: decedent's home (*n* = 2,680; 4.36%), nursing home or long-term care facility (*n* = 2,268; 3.69%), hospice facility (*n* = 1,123; 1.83%), and other or unknown locations (*n* = 719; 1.17%) (Supplementary Figure 4 and Supplementary Table S7).

### 3.9 Sensitivity analysis excluding the COVID-19 pandemic period (2020–2023)

Because several primary Joinpoint models (1999–2023) terminated within the pandemic period, the analyses were repeated using the same Joinpoint specifications but restricting the study period to 1999–2019. The significant post-2015 increase in the overall cohort (APC: 1.25%) was no longer statistically significant when 2020–2023 was excluded (APC: 0.29%, *p* = 0.80). Similarly, the late-period increase among women was no longer statistically significant after exclusion of 2020–2023, although the estimated APC increased from 1.98% to 2.71%. The corresponding APC estimates for NH Black adults, NH White adults, and adults aged 45–64 years were 1.93%, 0.40%, and 0.20%, respectively, and these late-period increases were also no longer statistically significant. The South showed the largest change: its significant 2017–2023 reversal (3.16%) was undetected in 1999–2019, where mortality continued declining significantly (−4.81%*). Strata already non-significant in the primary analysis (men, ages 65–85+, Northeast, Midwest, Hispanic/Latino adults, metropolitan/non-metropolitan areas) remained so, with comparable estimates; the West and NH Asian/Pacific Islander groups showed no reversal in either window. These findings suggest the late-period reversal's significance in several strata was partly pandemic-dependent, a caveat reflected in the Discussion (Supplementary Table S8, Supplementary Figure S5).

## 4 Discussion

The 25-year mortality trajectory for mesenteric ischemia comorbid with cardiovascular disease reflects a tension between advances in acute vascular intervention and the rising tide of metabolic burden in an aging population. The significant decline in early mortality may partly reflect the clinical adoption of endovascular revascularization, rapid computed tomography protocols, and more aggressive critical care support for sepsis (1). However, mortality increased from 2015 to 2023 (APC: 1.25%; 95% CI: 0.38 to 2.13), marking a reversal of the preceding declining trend. The confidence limit shows that this annual increase is relatively small when considering the data, which reflects the numbers between 0.4% and 2.1% (6). From a pathophysiological standpoint, the turning point might also indicate the enhanced influence of atherosclerotic load accumulation, low-flow conditions from heart failure, and coagulopathic issues that together contribute to problems associated with splanchnic perfusion (2).

The application of sensitivity analysis used in this study is necessary to contextualize the pattern of reversing mortality. While the pandemic years (2020-2023) were omitted from the analysis, the increase was much lower than the statistically significant increase we see otherwise in our results (APC: 0.29%; 95% CI −2.11 to 2.75 compared to a significant increase of APC 1.25%; 95% CI 0.38 to 2.13 in the previous analysis). Non-Hispanic Black adults and adults aged 45-64 experienced similar attenuation, as their rises in the later years were no longer statistically significant when 2020-2023 was excluded from the data. Similarly, the recent mortality increase observed in the Southern region was no longer detected when the pandemic years were excluded. The observed reversals therefore appear to have been partly influenced by the inclusion of the pandemic years. COVID-19-related hypercoagulability and disruptions in healthcare access remain plausible contributing mechanisms (14, 15); however, the sensitivity analysis cannot establish that COVID-19 itself directly caused these changes (Supplementary Table S8). The data reveal substantial disparities, with American Indian and Alaska Native populations experiencing the highest observed mortality rates. These disparities may reflect structural and healthcare-access factors, including the documented challenges affecting access to specialized care in some communities (16). Social and structural inequalities, rather than biology alone, influence these unfavorable results (17). The disparate impact in this group mirrors the higher prevalence of unmanaged diabetes and metabolic syndrome, conditions that facilitate atherosclerotic development (18). The same trend is observed among non-Hispanic Black individuals as mortality increased over the same period from 2016 to 2023 (APC=2.87%; 95% CI = 0.26-5.56). However, this increase was no longer statistically significant when the analysis was restricted to 1999–2019, suggesting that the observed reversal was partly influenced by the inclusion of the pandemic years. The wide confidence interval indicates an uncertain magnitude of the annual increase. Recent nationwide patient-level data provide additional context for these disparities. Bhatti et al. conducted a nationwide retrospective cohort study of patients hospitalized with acute mesenteric ischemia between 2016 and 2020. The researchers found that Native American patients had higher odds of in-hospital mortality compared with White patients, African American patients were less likely to undergo percutaneous vascular intervention. In addition, it was established that female patients had lower odds of in-hospital mortality than males (19). This trend may reveal some persistent disparities in the upstream management of cardiovascular risk factors (20). These trends may reflect differences in secondary prevention, socioeconomic conditions, healthcare accessibility, and logistical barriers affecting timely access to tertiary vascular centers (21). Asian and Pacific Islander communities have recorded lower mortality rates, but the broad racial groupings can hide the diversity in cardiovascular risks and the social factors affecting Asian subgroups (22).

Stratification by age highlights a distinct “survival effect” contributing to the substantial burden among the elderly. As improvements in cardiac care allow patients to survive initial myocardial infarctions and strokes, these individuals who survive progress to advanced mesenteric atherosclerosis (23). Consequently, the mortality burden shifts toward the oldest cohorts who possess the least physiological reserve (24). However, more critical is the appearance of evidence from the middle-aged cohort aged 45 to 64. Between 2013 and 2023, there was an upward trend in the mortality rate of people aged 45-64 (APC: 3.24%; 95% CI: 1.39-5.13). Notably, this increase was attenuated to an APC of 0.20% and was no longer statistically significant after exclusion of 2020–2023. According to the confidence interval, the annual increase that fits the data lies somewhere between about 1.4% and 5.1%, which supports the upward trend but indicates uncertainty regarding the precise magnitude of the increase. The growth in the death rate may partly reflect higher obesity and other cardiometabolic risks among younger populations (25). This shift predicts a caseload of younger, sicker patients with complex vascular anatomy. Harmantepe et al. have proposed a series of machine-learning models that can be used to estimate the risk of in-hospital mortality in patients diagnosed with acute mesenteric ischemia, identifying age, RDW, CRP, D-dimer, lactate, and creatinine as predictors of in-hospital mortality. These findings complement the present population-level results by highlighting the potential value of demographic and clinical laboratory variables for individual risk stratification (26). Our geographic analysis confirms a persistent “rural penalty” for mesenteric ischemia. The Southern region also demonstrated a recent increase from 2017 to 2023 (APC: 3.16%; 95% CI: 0.51 to 5.87). The relatively wide confidence interval indicates that, although the direction of the trend was upward, the precise magnitude of the annual increase remains uncertain. In the sensitivity analysis excluding the pandemic years, this reversal was not detected; instead, mortality continued to decline from 2009 to 2019 (APC: −4.81%; 95% CI: −6.02 to −3.58). The persistent high death rates in the Western region may partly reflect operational obstacles in referral pathways and interhospital transfer across long distances to centers offering advanced revascularization (8). Geographic maldistribution of vascular specialists may further contribute to reduced access to specialized vascular care in rural areas (27). The post-2015 data suggest that regionalization efforts did not sufficiently cover this gap, as national pandemic disruptions overwhelmed local infrastructure (28). The dynamic changes in state-level mortality likely indicate interdependence between local health policy and hospital system capabilities handling complex vascular emergencies (29, 30). Residents of rural areas continue to experience longer delays in emergency transport and have lower access to specialized care (31). Ultimately, these patterns occur against a broader background of substantial cardiovascular risk-factor burden and an aging population (32).

The high proportion of deaths occurring in inpatient medical facilities (85.65%) indicates that most decedents died after reaching an inpatient setting. This pattern warrants consideration of hospital-based factors such as diagnostic delay, timing of vascular evaluation, speed of revascularization, and acute management as potential hypotheses for future investigation. However, place-of-death data alone cannot determine whether mortality reflects prehospital delay, hospital processes, treatment decisions, disease severity at presentation, or a combination of these factors (31). Deaths occurring in nursing homes may disproportionately reflect a frail, institutionalized population with greater comorbidity and limited physiological reserve; however, place-of-death data cannot determine whether differences in treatment intensity contributed to these outcomes (33, 34). These findings indicate the imperatives of rapid triage systems and regionalized care networks, particularly as the population ages (3).

### 4.1 Public health and policy implications

This reversal in mortality, along with the unequal impact on various demographic as well as geographic groups, stresses the need for preventive measures and interventions to improve access to health care for those affected by such trends. Reversing these trends may require a shift from reactive treatment toward proactive surveillance. Clinical guidelines could support the mandate for screening for abdominal vascular disease in patients with known coronary or peripheral atherosclerosis (4). Early referral for mesenteric duplex ultrasound in patients reporting postprandial pain could prevent the progression to acute infarction (35). Such patients should be managed by a multidisciplinary team comprising vascular surgeons, gastroenterologists, and interventional radiologists (36). Furthermore, government policy initiatives must target the “rural penalty” by promoting vascular surgeons to practice in underserved regions and boosting financial support for telemedicine networks that can triage patients before transfer (37). For the high-risk American Indian and Black populations, resources must focus on culturally competent management of metabolic risk factors to halt the progression of vascular disease before it becomes surgical (16, 18, 21).

### 4.2 Limitations

We acknowledge several limitations inherent to administrative database research. Relying on death certificates introduces potential misclassification bias, as certifying physicians may list myocardial infarction as the primary cause while disregarding the underlying mesenteric ischemia. The absence of comprehensive clinical data pertaining to a patient prevents any form of adjustment being made for important prognostic factors, such as the length of time a patient lived with their symptoms, the time taken until presenting for treatment, the imaging results obtained, lab results, treatment used, specific techniques undertaken, degree of bowel death present, or results after surgery. Thus, CDC WONDER is incapable of determining whether temporal or demographic disparities in mortality imply differences in disease severity at presentation, treatment choice, or progressive condition. Because this was an observational, population-level mortality trend analysis based on death-certificate data, the findings identify temporal and subgroup associations but cannot establish causal relationships between cardiovascular comorbidity, clinical exposures, and mesenteric ischemia mortality. Urbanization classification ended in 2020, preventing analysis of how rural vs. metropolitan mortality diverged during the acute pandemic phase (2020-2021) and subsequent recovery period. The findings of this study are based on mortality data in America, therefore, the results of this study might not be directly generalizable to other populations which differ in the cardiovascular risk factor profile, health care system, and vascular care practices. Further studies are required to check whether the same patterns can be observed in different societies and settings. In addition, K55.0 and K55.1 were analyzed as a combined group. Because acute and chronic mesenteric ischemia have different clinical characteristics and management pathways, the present analysis cannot determine whether their individual mortality trends differed over time. Despite these constraints, the dataset's national scope provides a necessary high-level view of a rare but lethal condition that single-center studies cannot capture.

### 4.3 Future research directions

Further research needs to focus on patient-level variables that may contribute to the recent reversal in mortality trends, such as disease severity during presentation, diagnosis time, type of imaging, method of treatment, and time of revascularization. The combination of epidemiological data and clinical data from across the country will probably contribute to better understanding the reasons for demographic and geographic disparities in empirical findings. The integration of national epidemiological data with prediction models of patient level based on artificial intelligence and machine learning may allow for an improvement in the process of early risk assessment and further enhance the approach to the making of clinical decisions. Future studies should also assess whether these findings are reproducible in different populations and healthcare systems.

## 5 Conclusion

After more than two decades of decline, mortality from mesenteric ischemia with co-existing cardiovascular disease showed a reversal in its long-term declining pattern. The late-period increase occurred in the context of an aging population, cardiometabolic risk burden, and persistent racial and geographic disparities; however, these contextual factors were not directly measured and cannot be considered established causes of the observed trend. The findings support renewed attention to cardiovascular risk-factor prevention, timely recognition of mesenteric ischemia, and equitable access to specialized vascular care.

## Data Availability

The research is based on de-identified human death certificate data from the CDC WONDER database. https://wonder.cdc.gov/mcd.html.
