# Supplementary material for: Temporal trends and disparities in mesenteric ischemia–associated cardiovascular mortality among U.S. adults aged ≥45 years, 1999–2023
Source: Front Cardiovasc Med. 2026 Oct 9;13:1920942. doi: 10.3389/fcvm.2026.1920942 (PMC13690645; doi:10.3389/fcvm.2026.1920942)
Supplement: Supplementary file 1 [file Supplementaryfile1.docx]

**Supplementary Figure 1:** State-stratified age-adjusted mortality rates per 100,000 for Mesenteric Ischemia and Cardiovascular Disease-related mortality in United States adults aged ≥45 years, 1999-2020.
