# Supplementary material for: Temporal trends and disparities in mesenteric ischemia–associated cardiovascular mortality among U.S. adults aged ≥45 years, 1999–2023
Source: Front Cardiovasc Med. 2026 Oct 9;13:1920942. doi: 10.3389/fcvm.2026.1920942 (PMC13690645; doi:10.3389/fcvm.2026.1920942)
Supplement: Supplementary file 6 [file Table1.docx]

**Supplemental Table 1:** Age-Adjusted Mortality Rates (AAMR) and Deaths for Mesenteric Ischemia and Cardiovascular Disease-Related Mortality among United States Adults (Age 45+ years) stratified by Overall and Sex, 1999–2023.

| **Year** | **Overall** | | **Women** | | **Men** | |
| --- | --- | --- | --- | --- | --- | --- |
|  | **Deaths** | **AAMR (95% CI)** | **Deaths** | **AAMR (95% CI)** | **Deaths** | **AAMR (95% CI)** |
| **1999** | 4437 | 4.68 (4.54 - 4.81) | 2863 | 4.98 (4.79 - 5.16) | 1574 | 4.15 (3.94 - 4.36) |
| **2000** | 3989 | 4.14 (4.02 - 4.27) | 2548 | 4.36 (4.19 - 4.53) | 1441 | 3.77 (3.58 - 3.97) |
| **2001** | 3686 | 3.76 (3.64 - 3.89) | 2338 | 3.93 (3.77 - 4.09) | 1348 | 3.44 (3.25 - 3.63) |
| **2002** | 3379 | 3.41 (3.29 - 3.52) | 2132 | 3.57 (3.42 - 3.73) | 1247 | 3.15 (2.97 - 3.33) |
| **2003** | 3253 | 3.22 (3.11 - 3.33) | 2037 | 3.39 (3.24 - 3.54) | 1216 | 2.99 (2.82 - 3.16) |
| **2004** | 2923 | 2.84 (2.74 - 2.95) | 1819 | 2.98 (2.84 - 3.12) | 1104 | 2.65 (2.49 - 2.81) |
| **2005** | 2739 | 2.65 (2.55 - 2.75) | 1697 | 2.74 (2.60 - 2.87) | 1042 | 2.40 (2.25 - 2.55) |
| **2006** | 2616 | 2.45 (2.36 - 2.55) | 1603 | 2.55 (2.42 - 2.67) | 1013 | 2.28 (2.14 - 2.42) |
| **2007** | 2496 | 2.32 (2.23 - 2.41) | 1544 | 2.37 (2.25 - 2.49) | 952 | 2.12 (1.99 - 2.26) |
| **2008** | 2393 | 2.19 (2.10 - 2.28) | 1463 | 2.28 (2.16 - 2.39) | 930 | 2.04 (1.90 - 2.17) |
| **2009** | 2249 | 1.98 (1.90 - 2.07) | 1354 | 2.04 (1.93 - 2.15) | 895 | 1.86 (1.73 - 1.98) |
| **2010** | 2078 | 1.80 (1.72 - 1.88) | 1306 | 1.93 (1.82 - 2.03) | 772 | 1.57 (1.46 - 1.69) |
| **2011** | 2126 | 1.81 (1.73 - 1.88) | 1254 | 1.83 (1.73 - 1.94) | 872 | 1.75 (1.63 - 1.87) |
| **2012** | 1959 | 1.61 (1.53 - 1.68) | 1194 | 1.70 (1.60 - 1.80) | 765 | 1.47 (1.36 - 1.57) |
| **2013** | 1866 | 1.48 (1.41 - 1.54) | 1106 | 1.55 (1.45 - 1.64) | 760 | 1.41 (1.31 - 1.51) |
| **2014** | 1814 | 1.43 (1.36 - 1.50) | 1096 | 1.48 (1.39 - 1.57) | 718 | 1.29 (1.20 - 1.39) |
| **2015** | 1748 | 1.32 (1.25 - 1.38) | 1053 | 1.41 (1.33 - 1.50) | 695 | 1.21 (1.12 - 1.30) |
| **2016** | 1804 | 1.36 (1.30 - 1.42) | 1034 | 1.36 (1.27 - 1.44) | 770 | 1.30 (1.20 - 1.39) |
| **2017** | 1823 | 1.30 (1.24 - 1.36) | 1031 | 1.32 (1.24 - 1.40) | 792 | 1.30 (1.21 - 1.40) |
| **2018** | 1834 | 1.31 (1.25 - 1.37) | 1125 | 1.40 (1.31 - 1.48) | 709 | 1.17 (1.08 - 1.26) |
| **2019** | 1941 | 1.35 (1.29 - 1.41) | 1141 | 1.42 (1.33 - 1.50) | 800 | 1.27 (1.18 - 1.36) |
| **2020** | 1943 | 1.34 (1.28 - 1.40) | 1115 | 1.34 (1.26 - 1.42) | 828 | 1.27 (1.18 - 1.35) |
| **2021** | 2118 | 1.46 (1.40 - 1.52) | 1196 | 1.50 (1.42 - 1.59) | 922 | 1.44 (1.34 - 1.53) |
| **2022** | 2141 | 1.41 (1.35 - 1.47) | 1251 | 1.48 (1.40 - 1.56) | 890 | 1.30 (1.21 - 1.38) |
| **2023** | 2188 | 1.47 (1.41 - 1.53) | 1283 | 1.50 (1.42 - 1.58) | 905 | 1.32 (1.23 - 1.41) |
| **Total/Average** | 61543 | 2.16 (2.08 - 2.25) | 37583 | 2.26 (2.14 - 2.37) | 23960 | 2.00 (1.87 - 2.12) |
