# Supplementary material for: Temporal trends and disparities in mesenteric ischemia–associated cardiovascular mortality among U.S. adults aged ≥45 years, 1999–2023
Source: Front Cardiovasc Med. 2026 Oct 9;13:1920942. doi: 10.3389/fcvm.2026.1920942 (PMC13690645; doi:10.3389/fcvm.2026.1920942)
Supplement: Supplementary file 7 [file Table2.docx]

**Supplemental Table 2:** Age-Adjusted Mortality Rates (AAMR) and Deaths for Mesenteric Ischemia and Cardiovascular Disease -Related Mortality among United States Adults (Age 45+ years) stratified by Race, 1999–2023.

| Year | NH American Indian or Alaska Native | | NH Asian or Pacific Islander | | NH Black or African American | | NH White | | Hispanic or Latino | |
| --- | --- | --- | --- | --- | --- | --- | --- | --- | --- | --- |
|  | **Deaths** | **AAMR (95% CI)** | **Deaths** | **AAMR (95% CI)** | **Deaths** | **AAMR (95% CI)** | **Deaths** | **AAMR (95% CI)** | **Deaths** | **AAMR (95% CI)** |
| **1999** | 17 | Unreliable (2.52 to 7.43) | 54 | 2.62 (1.95 to 3.46) | 412 | 5.21 (4.71 to 5.72) | 210 | 4.86 (4.18 to 5.54) | 3737 | 4.63 (4.48 to 4.78) |
| **2000** | 26 | 5.75 (3.69 to 8.56) | 40 | 1.97 (1.39 to 2.71) | 371 | 4.63 (4.16 to 5.11) | 186 | 4.30 (3.66 to 4.93) | 3354 | 4.10 (3.96 to 4.23) |
| **2001** | 17 | Unreliable (2.72 to 7.74) | 52 | 2.12 (1.57 to 2.81) | 330 | 4.01 (3.57 to 4.45) | 156 | 3.32 (2.78 to 3.85) | 3124 | 3.80 (3.67 to 3.93) |
| **2002** | 21 | 5.61 (3.43 to 8.66) | 44 | 1.69 (1.21 to 2.29) | 293 | 3.53 (3.12 to 3.94) | 153 | 2.93 (2.45 to 3.42) | 2859 | 3.44 (3.32 to 3.57) |
| **2003** | 20 | 4.28 (2.53 to 6.76) | 43 | 1.61 (1.15 to 2.19) | 277 | 3.13 (2.76 to 3.51) | 146 | 2.66 (2.22 to 3.11) | 2764 | 3.27 (3.15 to 3.39) |
| **2004** | 12 | Unreliable (1.26 to 4.53) | 40 | 1.49 (1.06 to 2.05) | 271 | 3.15 (2.77 to 3.54) | 140 | 2.44 (2.02 to 2.85) | 2456 | 2.92 (2.80 to 3.03) |
| **2005** | 18 | Unreliable (2.15 to 6.11) | 45 | 1.58 (1.14 to 2.13) | 239 | 2.66 (2.32 to 3.00) | 161 | 2.60 (2.18 to 3.01) | 2268 | 2.65 (2.54 to 2.76) |
| **2006** | 15 | Unreliable (1.60 to 5.14) | 41 | 1.41 (1.01 to 1.92) | 217 | 2.41 (2.08 to 2.73) | 127 | 1.99 (1.63 to 2.34) | 2214 | 2.54 (2.44 to 2.65) |
| **2007** | 14 | Unreliable (1.29 to 4.14) | 43 | 1.26 (0.90 to 1.71) | 209 | 2.21 (1.90 to 2.51) | 128 | 1.93 (1.58 to 2.27) | 2099 | 2.37 (2.27 to 2.47) |
| **2008** | 13 | Unreliable (1.35 to 4.57) | 48 | 1.28 (0.94 to 1.72) | 208 | 2.12 (1.83 to 2.42) | 172 | 2.47 (2.09 to 2.85) | 1950 | 2.16 (2.06 to 2.26) |
| **2009** | 20 | 3.64 (2.16 to 5.75) | 47 | 1.25 (0.91 to 1.67) | 206 | 2.06 (1.77 to 2.35) | 137 | 1.85 (1.53 to 2.17) | 1834 | 2.02 (1.93 to 2.11) |
| **2010** | 15 | Unreliable (1.42 to 4.56) | 33 | 0.83 (0.56 to 1.18) | 200 | 1.90 (1.63 to 2.17) | 145 | 1.84 (1.53 to 2.16) | 1680 | 1.80 (1.72 to 1.89) |
| **2011** | 20 | 3.22 (1.91 to 5.10) | 44 | 1.04 (0.75 to 1.40) | 184 | 1.75 (1.49 to 2.01) | 122 | 1.46 (1.20 to 1.73) | 1753 | 1.85 (1.76 to 1.93) |
| **2012** | 22 | 3.27 (2.00 to 5.05) | 35 | 0.78 (0.54 to 1.09) | 162 | 1.48 (1.24 to 1.71) | 121 | 1.39 (1.14 to 1.65) | 1611 | 1.66 (1.58 to 1.74) |
| **2013** | 17 | Unreliable (1.32 to 3.89) | 33 | 0.67 (0.45 to 0.95) | 195 | 1.71 (1.46 to 1.96) | 122 | 1.33 (1.09 to 1.57) | 1494 | 1.52 (1.44 to 1.59) |
| **2014** | 17 | Unreliable (1.35 to 3.83) | 26 | 0.46 (0.30 to 0.69) | 174 | 1.48 (1.25 to 1.70) | 130 | 1.28 (1.06 to 1.51) | 1467 | 1.46 (1.39 to 1.54) |
| **2015** | 20 | 2.59 (1.54 to 4.10) | 32 | 0.61 (0.42 to 0.87) | 168 | 1.40 (1.18 to 1.62) | 114 | 1.06 (0.86 to 1.26) | 1405 | 1.36 (1.29 to 1.43) |
| **2016** | 19 | Unreliable (1.50 to 3.99) | 47 | 0.81 (0.59 to 1.08) | 151 | 1.17 (0.98 to 1.36) | 133 | 1.28 (1.06 to 1.51) | 1446 | 1.43 (1.35 to 1.50) |
| **2017** | 14 | Unreliable (1.01 to 3.25) | 47 | 0.72 (0.53 to 0.97) | 177 | 1.36 (1.15 to 1.56) | 152 | 1.20 (1.00 to 1.40) | 1429 | 1.37 (1.30 to 1.44) |
| **2018** | 15 | Unreliable (1.12 to 3.42) | 38 | 0.55 (0.39 to 0.76) | 185 | 1.34 (1.14 to 1.54) | 145 | 1.24 (1.03 to 1.45) | 1445 | 1.34 (1.27 to 1.41) |
| **2019** | 18 | Unreliable (1.22 to 3.35) | 41 | 0.57 (0.41 to 0.77) | 191 | 1.35 (1.15 to 1.54) | 159 | 1.25 (1.05 to 1.45) | 1528 | 1.39 (1.32 to 1.46) |
| **2020** | 17 | Unreliable (0.95 to 2.69) | 37 | 0.48 (0.33 to 0.67) | 194 | 1.31 (1.12 to 1.50) | 157 | 1.20 (1.01 to 1.39) | 1534 | 1.40 (1.32 to 1.47) |
| **2021** | 21 | 2.40 (1.46 to 3.70) | 47 | 0.66 (0.49 to 0.88) | 241 | 1.69 (1.47 to 1.92) | 203 | 1.46 (1.25 to 1.67) | 1589 | 1.48 (1.41 to 1.55) |
| **2022** | 24 | 2.62 (1.66 to 3.93) | 51 | 0.68 (0.50 to 0.90) | 215 | 1.40 (1.21 to 1.60) | 199 | 1.37 (1.18 to 1.57) | 1641 | 1.52 (1.45 to 1.60) |
| **2023** | 20 | 2.18 (1.32 to 3.41) | 56 | 0.67 (0.51 to 0.88) | 236 | 1.57 (1.36 to 1.77) | 200 | 1.39 (1.20 to 1.59) | 1664 | 1.51 (1.43 to 1.58) |
| **Total/Average** | 452 | 3.56 (1.78 to 4.95) | 1064 | 1.11 (0.80 to 1.51) | 5706 | 2.24 (1.95 to 2.53) | 3818 | 2.00 (1.68 to 2.33) | 50345 | 2.20 (2.11 to 2.29) |
