# Supplementary material for: Temporal trends and disparities in mesenteric ischemia–associated cardiovascular mortality among U.S. adults aged ≥45 years, 1999–2023
Source: Front Cardiovasc Med. 2026 Oct 9;13:1920942. doi: 10.3389/fcvm.2026.1920942 (PMC13690645; doi:10.3389/fcvm.2026.1920942)
Supplement: Supplementary file 8 [file Table3.docx]

**Supplemental Table 3:** Crude Mortality Rates (CMR) and Deaths for Mesenteric Ischemia and Cardiovascular Disease -Related Mortality among United States Adults (Age 45+ years) stratified by Age group, 1999–2023.

| **Year** | **45-64 Years** | | **65-85+ Years** | |
| --- | --- | --- | --- | --- |
|  | **Deaths** | **CMR (95% CI)** | **Deaths** | **CMR (95% CI)** |
| **1999** | 594 | 0.98 (0.91 - 1.06) | 3843 | 11.04 (10.69 - 11.39) |
| **2000** | 517 | 0.83 (0.76 - 0.91) | 3472 | 9.92 (9.59 - 10.25) |
| **2001** | 509 | 0.79 (0.72 - 0.86) | 3177 | 9.00 (8.69 - 9.32) |
| **2002** | 468 | 0.70 (0.64 - 0.77) | 2911 | 8.19 (7.90 - 8.49) |
| **2003** | 486 | 0.71 (0.64 - 0.77) | 2767 | 7.72 (7.43 - 8.00) |
| **2004** | 423 | 0.60 (0.54 - 0.65) | 2500 | 6.91 (6.63 - 7.18) |
| **2005** | 420 | 0.57 (0.52 - 0.63) | 2319 | 6.33 (6.07 - 6.58) |
| **2006** | 427 | 0.57 (0.51 - 0.62) | 2189 | 5.89 (5.64 - 6.14) |
| **2007** | 406 | 0.53 (0.48 - 0.58) | 2090 | 5.53 (5.29 - 5.76) |
| **2008** | 409 | 0.52 (0.47 - 0.57) | 1984 | 5.12 (4.89 - 5.34) |
| **2009** | 371 | 0.46 (0.42 - 0.51) | 1878 | 4.74 (4.53 - 4.95) |
| **2010** | 388 | 0.48 (0.43 - 0.52) | 1690 | 4.20 (4.00 - 4.40) |
| **2011** | 350 | 0.42 (0.38 - 0.47) | 1776 | 4.29 (4.09 - 4.49) |
| **2012** | 364 | 0.44 (0.39 - 0.48) | 1595 | 3.70 (3.52 - 3.88) |
| **2013** | 335 | 0.40 (0.36 - 0.45) | 1531 | 3.42 (3.25 - 3.60) |
| **2014** | 353 | 0.42 (0.38 - 0.47) | 1461 | 3.16 (3.00 - 3.32) |
| **2015** | 317 | 0.38 (0.34 - 0.42) | 1431 | 3.00 (2.84 - 3.15) |
| **2016** | 363 | 0.43 (0.39 - 0.48) | 1441 | 2.93 (2.78 - 3.08) |
| **2017** | 369 | 0.44 (0.39 - 0.48) | 1454 | 2.86 (2.71 - 3.01) |
| **2018** | 327 | 0.39 (0.35 - 0.43) | 1507 | 2.87 (2.73 - 3.02) |
| **2019** | 370 | 0.44 (0.40 - 0.49) | 1571 | 2.91 (2.76 - 3.05) |
| **2020** | 437 | 0.53 (0.48 - 0.58) | 1506 | 2.71 (2.57 - 2.84) |
| **2021** | 422 | 0.51 (0.46 - 0.55) | 1696 | 3.04 (2.89 - 3.18) |
| **2022** | 432 | 0.52 (0.47 - 0.57) | 1709 | 2.96 (2.82 - 3.10) |
| **2023** | 426 | 0.52 (0.47 - 0.57) | 1762 | 2.97 (2.84 - 3.11) |
| **Total/Average** | 10283 | 0.54 (0.49 - 0.60) | 51260 | 5.02 (4.81 - 5.23) |
