# Supplementary material for: Temporal trends and disparities in mesenteric ischemia–associated cardiovascular mortality among U.S. adults aged ≥45 years, 1999–2023
Source: Front Cardiovasc Med. 2026 Oct 9;13:1920942. doi: 10.3389/fcvm.2026.1920942 (PMC13690645; doi:10.3389/fcvm.2026.1920942)
Supplement: Supplementary file 9 [file Table4.docx]

**Supplemental Table 4:** Age-Adjusted Mortality Rates (AAMR) and Deaths for Mesenteric Ischemia and Cardiovascular Disease -Related Mortality among United States Adults (Age 45+ years) stratified by Urbanization, 1999–2020.

| **Year** | **Metropolitan** | | **Non-metropolitan** | |
| --- | --- | --- | --- | --- |
|  | **Deaths** | **AAMR (95% CI)** | **Deaths** | **AAMR (95% CI)** |
| **1999** | 3526 | 4.56 (4.41 - 4.71) | 911 | 5.03 (4.70 - 5.36) |
| **2000** | 3205 | 4.11 (3.97 - 4.25) | 784 | 4.28 (3.98 - 4.58) |
| **2001** | 2939 | 3.73 (3.60 - 3.87) | 747 | 4.03 (3.74 - 4.32) |
| **2002** | 2622 | 3.26 (3.14 - 3.39) | 757 | 4.08 (3.79 - 4.38) |
| **2003** | 2553 | 3.13 (3.00 - 3.25) | 700 | 3.70 (3.43 - 3.98) |
| **2004** | 2275 | 2.74 (2.63 - 2.85) | 648 | 3.41 (3.15 - 3.67) |
| **2005** | 2167 | 2.56 (2.46 - 2.67) | 572 | 3.00 (2.75 - 3.24) |
| **2006** | 2077 | 2.40 (2.30 - 2.51) | 539 | 2.77 (2.54 - 3.01) |
| **2007** | 1966 | 2.24 (2.14 - 2.34) | 530 | 2.70 (2.47 - 2.93) |
| **2008** | 1869 | 2.09 (1.99 - 2.18) | 524 | 2.62 (2.40 - 2.85) |
| **2009** | 1743 | 1.87 (1.78 - 1.96) | 506 | 2.49 (2.27 - 2.71) |
| **2010** | 1637 | 1.72 (1.64 - 1.81) | 441 | 2.15 (1.95 - 2.35) |
| **2011** | 1667 | 1.72 (1.63 - 1.80) | 459 | 2.20 (2.00 - 2.40) |
| **2012** | 1510 | 1.49 (1.41 - 1.57) | 449 | 2.11 (1.91 - 2.31) |
| **2013** | 1464 | 1.43 (1.35 - 1.50) | 402 | 1.87 (1.69 - 2.06) |
| **2014** | 1455 | 1.40 (1.32 - 1.47) | 359 | 1.64 (1.47 - 1.81) |
| **2015** | 1369 | 1.27 (1.21 - 1.34) | 379 | 1.71 (1.54 - 1.89) |
| **2016** | 1429 | 1.29 (1.22 - 1.35) | 375 | 1.64 (1.47 - 1.81) |
| **2017** | 1437 | 1.25 (1.19 - 1.32) | 386 | 1.67 (1.50 - 1.84) |
| **2018** | 1441 | 1.22 (1.16 - 1.29) | 393 | 1.66 (1.50 - 1.83) |
| **2019** | 1519 | 1.27 (1.21 - 1.34) | 422 | 1.77 (1.60 - 1.95) |
| **2020** | 1508 | 1.21 (1.15 - 1.27) | 435 | 1.80 (1.63 - 1.97) |
| **Total/Average** | 43378 | 2.18 (2.09 - 2.27) | 11718 | 2.65 (2.43 - 2.88) |
