# Supplementary material for: Temporal trends and disparities in mesenteric ischemia–associated cardiovascular mortality among U.S. adults aged ≥45 years, 1999–2023
Source: Front Cardiovasc Med. 2026 Oct 9;13:1920942. doi: 10.3389/fcvm.2026.1920942 (PMC13690645; doi:10.3389/fcvm.2026.1920942)
Supplement: Supplementary file 10 [file Table5.docx]

**Supplemental Table 5:** Age-Adjusted Mortality Rates (AAMR) and Deaths for Mesenteric Ischemia and Cardiovascular Disease -Related Mortality among United States Adults (Age 45+ years) stratified by Census region, 1999–2023.

| **Year** | **Northeast** | | **Midwest** | | **South** | | **West** | |
| --- | --- | --- | --- | --- | --- | --- | --- | --- |
|  | **Deaths** | **AAMR (95% CI)** | **Deaths** | **AAMR (95% CI)** | **Deaths** | **AAMR (95% CI)** | **Deaths** | **AAMR (95% CI)** |
| **1999** | 865 | 4.25 (3.97 to 4.54) | 1045 | 4.58 (4.30 to 4.85) | 1654 | 4.95 (4.71 to 5.18) | 873 | 4.67 (4.36 to 4.98) |
| **2000** | 747 | 3.63 (3.37 to 3.89) | 864 | 3.77 (3.52 to 4.03) | 1531 | 4.54 (4.32 to 4.77) | 847 | 4.46 (4.16 to 4.76) |
| **2001** | 687 | 3.34 (3.09 to 3.59) | 909 | 3.90 (3.65 to 4.15) | 1236 | 3.62 (3.42 to 3.83) | 854 | 4.37 (4.07 to 4.66) |
| **2002** | 597 | 2.85 (2.62 to 3.08) | 841 | 3.60 (3.35 to 3.84) | 1234 | 3.53 (3.33 to 3.72) | 707 | 3.55 (3.29 to 3.81) |
| **2003** | 595 | 2.82 (2.59 to 3.05) | 740 | 3.12 (2.90 to 3.35) | 1194 | 3.34 (3.15 to 3.52) | 724 | 3.52 (3.26 to 3.78) |
| **2004** | 494 | 2.33 (2.13 to 2.54) | 763 | 3.18 (2.95 to 3.40) | 1075 | 2.97 (2.79 to 3.15) | 591 | 2.81 (2.58 to 3.04) |
| **2005** | 523 | 2.41 (2.21 to 2.62) | 633 | 2.63 (2.43 to 2.84) | 952 | 2.56 (2.40 to 2.73) | 631 | 2.93 (2.70 to 3.16) |
| **2006** | 481 | 2.20 (2.00 to 2.39) | 596 | 2.42 (2.23 to 2.62) | 928 | 2.45 (2.29 to 2.61) | 611 | 2.76 (2.54 to 2.98) |
| **2007** | 462 | 2.09 (1.90 to 2.28) | 615 | 2.45 (2.25 to 2.64) | 872 | 2.24 (2.09 to 2.38) | 547 | 2.41 (2.21 to 2.61) |
| **2008** | 449 | 2.02 (1.83 to 2.20) | 557 | 2.17 (1.99 to 2.36) | 863 | 2.15 (2.01 to 2.29) | 524 | 2.22 (2.03 to 2.41) |
| **2009** | 404 | 1.80 (1.62 to 1.98) | 572 | 2.20 (2.02 to 2.38) | 753 | 1.86 (1.73 to 1.99) | 520 | 2.15 (1.96 to 2.33) |
| **2010** | 370 | 1.61 (1.44 to 1.77) | 502 | 1.91 (1.74 to 2.08) | 746 | 1.79 (1.66 to 1.92) | 460 | 1.87 (1.70 to 2.04) |
| **2011** | 361 | 1.54 (1.38 to 1.70) | 522 | 1.93 (1.76 to 2.09) | 742 | 1.73 (1.60 to 1.85) | 501 | 1.95 (1.78 to 2.12) |
| **2012** | 329 | 1.38 (1.23 to 1.53) | 478 | 1.72 (1.56 to 1.87) | 678 | 1.54 (1.42 to 1.66) | 474 | 1.82 (1.65 to 1.99) |
| **2013** | 317 | 1.30 (1.16 to 1.45) | 442 | 1.59 (1.44 to 1.74) | 657 | 1.42 (1.32 to 1.53) | 450 | 1.69 (1.53 to 1.85) |
| **2014** | 301 | 1.26 (1.12 to 1.40) | 424 | 1.52 (1.37 to 1.67) | 658 | 1.43 (1.32 to 1.54) | 431 | 1.54 (1.39 to 1.69) |
| **2015** | 273 | 1.08 (0.95 to 1.20) | 398 | 1.42 (1.27 to 1.56) | 663 | 1.39 (1.28 to 1.49) | 414 | 1.45 (1.31 to 1.59) |
| **2016** | 291 | 1.16 (1.02 to 1.29) | 413 | 1.42 (1.28 to 1.55) | 616 | 1.24 (1.14 to 1.34) | 484 | 1.62 (1.47 to 1.77) |
| **2017** | 330 | 1.27 (1.13 to 1.41) | 401 | 1.32 (1.19 to 1.46) | 618 | 1.20 (1.11 to 1.30) | 474 | 1.56 (1.41 to 1.70) |
| **2018** | 299 | 1.15 (1.01 to 1.28) | 408 | 1.33 (1.20 to 1.47) | 650 | 1.23 (1.13 to 1.32) | 477 | 1.54 (1.40 to 1.68) |
| **2019** | 356 | 1.33 (1.19 to 1.47) | 442 | 1.43 (1.30 to 1.57) | 654 | 1.18 (1.09 to 1.28) | 489 | 1.51 (1.37 to 1.64) |
| **2020** | 297 | 1.10 (0.97 to 1.22) | 444 | 1.38 (1.25 to 1.51) | 714 | 1.29 (1.19 to 1.39) | 488 | 1.50 (1.36 to 1.63) |
| **2021** | 310 | 1.15 (1.02 to 1.28) | 475 | 1.59 (1.44 to 1.73) | 776 | 1.42 (1.32 to 1.53) | 557 | 1.73 (1.59 to 1.88) |
| **2022** | 329 | 1.15 (1.03 to 1.28) | 494 | 1.57 (1.43 to 1.71) | 792 | 1.37 (1.27 to 1.46) | 526 | 1.58 (1.45 to 1.72) |
| **2023** | 367 | 1.30 (1.16 to 1.43) | 482 | 1.50 (1.36 to 1.63) | 783 | 1.36 (1.26 to 1.45) | 556 | 1.64 (1.51 to 1.78) |
| **Total/Average** | 10834 | 1.90 (1.73 to 2.07) | 14460 | 2.23 (2.05 to 2.40) | 22039 | 2.15 (2.01 to 2.29) | 14210 | 2.35 (2.16 to 2.54) |
