# Supplementary material for: Temporal trends and disparities in mesenteric ischemia–associated cardiovascular mortality among U.S. adults aged ≥45 years, 1999–2023
Source: Front Cardiovasc Med. 2026 Oct 9;13:1920942. doi: 10.3389/fcvm.2026.1920942 (PMC13690645; doi:10.3389/fcvm.2026.1920942)
Supplement: Supplementary file 11 [file Table6.docx]

**Supplemental Table 6:** Age-Adjusted Mortality Rates (AAMR) and Deaths for Mesenteric Ischemia and Cardiovascular Disease -Related Mortality among United States Adults (Age 45+ years) stratified by State, 1999–2023.

| **State** | **1999-2020** | | **2021-2023** | | **1999-2023** |
| --- | --- | --- | --- | --- | --- |
|  | **Deaths** | **AAMR (95% CI)** | **Deaths** | **AAMR (95% CI)** | **Deaths** |
| **Alabama** | 977 | 2.40 (2.25-2.55) | 87 | 1.23 (0.98 - 1.53) | 1064 |
| **Alaska** | 101 | 2.99 (2.37-3.60) | 15 | Unreliable (1.04 - 3.34) | 116 |
| **Arizona** | 871 | 1.59 (1.49-1.70) | 146 | 1.38 (1.15 - 1.61) | 1017 |
| **Arkansas** | 613 | 2.41 (2.21-2.60) | 79 | 1.94 (1.53 - 2.43) | 692 |
| **California** | 6714 | 2.41 (2.35-2.47) | 684 | 1.44 (1.33 - 1.55) | 7398 |
| **Colorado** | 743 | 2.12 (1.96-2.27) | 132 | 2.00 (1.66 - 2.35) | 875 |
| **Connecticut** | 609 | 1.82 (1.67-1.96) | 43 | 0.82 (0.59 - 1.10) | 652 |
| **Delaware** | 141 | 1.78 (1.48-2.08) | 20 | 1.33 (0.80 - 2.08) | 161 |
| **District of Columbia** | 108 | 2.36 (1.91-2.81) | ----- | ----- | 108* |
| **Florida** | 3232 | 1.61 (1.56-1.67) | 401 | 1.12 (1.01 - 1.24) | 3633 |
| **Georgia** | 1303 | 1.98 (1.87-2.09) | 142 | 1.13 (0.94 - 1.32) | 1445 |
| **Hawaii** | 190 | 1.53 (1.31-1.74) | 21 | 0.83 (0.52 - 1.27) | 211 |
| **Idaho** | 350 | 2.89 (2.58-3.19) | 48 | 2.09 (1.53 - 2.78) | 398 |
| **Illinois** | 2031 | 1.94 (1.85-2.02) | 171 | 1.01 (0.86 - 1.17) | 2202 |
| **Indiana** | 1387 | 2.58 (2.44-2.72) | 169 | 1.91 (1.62 - 2.20) | 1556 |
| **Iowa** | 632 | 2.12 (1.95-2.29) | 66 | 1.48 (1.14 - 1.90) | 698 |
| **Kansas** | 545 | 2.25 (2.06-2.44) | 63 | 1.64 (1.25 - 2.10) | 608 |
| **Kentucky** | 948 | 2.65 (2.48-2.82) | 92 | 1.51 (1.21 - 1.86) | 1040 |
| **Louisiana** | 693 | 1.93 (1.79-2.07) | 64 | 1.03 (0.78 - 1.32) | 757 |
| **Maine** | 330 | 2.48 (2.21-2.75) | 50 | 2.00 (1.48 - 2.64) | 380 |
| **Maryland** | 793 | 1.76 (1.63-1.88) | 132 | 1.57 (1.30 - 1.84) | 925 |
| **Massachusetts** | 994 | 1.65 (1.55-1.75) | 109 | 1.13 (0.91 - 1.34) | 1103 |
| **Michigan** | 1956 | 2.28 (2.18-2.38) | 207 | 1.47 (1.27 - 1.67) | 2163 |
| **Minnesota** | 931 | 2.05 (1.92-2.18) | 180 | 2.37 (2.02 - 2.72) | 1111 |
| **Mississippi** | 471 | 1.96 (1.78-2.13) | 47 | 1.24 (0.91 - 1.66) | 518 |
| **Missouri** | 1222 | 2.31 (2.18-2.45) | 125 | 1.46 (1.20 - 1.72) | 1347 |
| **Montana** | 199 | 2.16 (1.86-2.46) | 22 | 1.29 (0.80 - 1.97) | 221 |
| **Nebraska** | 392 | 2.42 (2.18-2.66) | 39 | 1.49 (1.05 - 2.05) | 431 |
| **Nevada** | 334 | 1.70 (1.51-1.88) | 89 | 2.21 (1.76 - 2.73) | 423 |
| **New Hampshire** | 279 | 2.41 (2.12-2.69) | 35 | 1.73 (1.20 - 2.41) | 314 |
| **New Jersey** | 1222 | 1.57 (1.48-1.66) | 118 | 0.92 (0.75 - 1.09) | 1340 |
| **New Mexico** | 339 | 2.06 (1.84-2.28) | 40 | 1.34 (0.94 - 1.85) | 379 |
| **New York** | 3299 | 1.93 (1.86-2.00) | 339 | 1.20 (1.07 - 1.33) | 3638 |
| **North Carolina** | 1654 | 2.15 (2.05-2.26) | 201 | 1.41 (1.22 - 1.61) | 1855 |
| **North Dakota** | 150 | 2.25 (1.89-2.62) | 15 | Unreliable (0.89 - 2.75) | 165 |
| **Ohio** | 2479 | 2.39 (2.29-2.48) | 236 | 1.47 (1.28 - 1.66) | 2715 |
| **Oklahoma** | 905 | 2.87 (2.68-3.06) | 114 | 2.20 (1.79 - 2.61) | 1019 |
| **Oregon** | 886 | 2.60 (2.43-2.77) | 151 | 2.51 (2.10 - 2.92) | 1037 |
| **Pennsylvania** | 2665 | 2.12 (2.04-2.20) | 262 | 1.33 (1.17 - 1.49) | 2927 |
| **Rhode Island** | 238 | 2.30 (2.01-2.60) | 26 | 1.58 (1.02 - 2.33) | 264 |
| **South Carolina** | 939 | 2.37 (2.22-2.53) | 151 | 1.99 (1.67 - 2.32) | 1090 |
| **South Dakota** | 152 | 1.97 (1.65-2.29) | 31 | 2.44 (1.65 - 3.49) | 183 |
| **Tennessee** | 1448 | 2.73 (2.59-2.87) | 181 | 1.96 (1.67 - 2.25) | 1629 |
| **Texas** | 4000 | 2.41 (2.33-2.48) | 451 | 1.42 (1.28 - 1.55) | 4451 |
| **Utah** | 312 | 1.97 (1.75-2.19) | 45 | 1.45 (1.05 - 1.95) | 357 |
| **Vermont** | 192 | 3.24 (2.78-3.70) | 24 | 2.28 (1.46 - 3.40) | 216 |
| **Virginia** | 981 | 1.62 (1.52-1.72) | 141 | 1.25 (1.04 - 1.46) | 1122 |
| **Washington** | 1409 | 2.66 (2.52-2.80) | 235 | 2.39 (2.08 - 2.70) | 1644 |
| **West Virginia** | 482 | 2.64 (2.41-2.88) | 41 | 1.43 (1.02 - 1.95) | 523 |
| **Wisconsin** | 1132 | 2.24 (2.11-2.37) | 149 | 1.81 (1.52 - 2.11) | 1281 |
| **Wyoming** | 123 | 2.82 (2.31-3.32) | 11 | Unreliable (0.75 - 2.69) | 134 |
| **Total** | 55096 | ----- | 6337 | ----- | **61428** |
