# Supplementary material for: Temporal trends and disparities in mesenteric ischemia–associated cardiovascular mortality among U.S. adults aged ≥45 years, 1999–2023
Source: Front Cardiovasc Med. 2026 Oct 9;13:1920942. doi: 10.3389/fcvm.2026.1920942 (PMC13690645; doi:10.3389/fcvm.2026.1920942)
Supplement: Supplementary file 12 [file Table7.docx]

**Supplemental Table 7:** Percentage distribution of place of death in the United States, 1999–2023, highlighting Mesenteric Ischemia and Cardiovascular Disease–related mortality

| **Place of Death** | **Deaths** | **%Total** |
| --- | --- | --- |
| **Medical Facility - Inpatient** | 52702 | 85.65% |
| **Decedent's home** | 2680 | 4.36% |
| **Nursing home/long term care** | 2268 | 3.69% |
| **Medical Facility - Outpatient or ER** | 1904 | 3.09% |
| **Hospice facility** | 1123 | 1.83% |
| **Other** | 571 | 0.93% |
| **Place of death unknown** | 148 | 0.24% |
| **Medical Facility - Status unknown** | 92 | 0.15% |
| **Medical Facility - Dead on Arrival** | 46 | 0.07% |
| **Total** | **61534** | **100%** |
