# Supplementary material for: Temporal trends and disparities in mesenteric ischemia–associated cardiovascular mortality among U.S. adults aged ≥45 years, 1999–2023
Source: Front Cardiovasc Med. 2026 Oct 9;13:1920942. doi: 10.3389/fcvm.2026.1920942 (PMC13690645; doi:10.3389/fcvm.2026.1920942)
Supplement: Supplementary file 13 [file Table8.docx]

**Supplementary Table 8. Primary analysis (1999–2023) vs. sensitivity analysis (1999–2019, pandemic years excluded): final Joinpoint segment for each stratum.**

| **Cohort** | **Primary segment** | **Primary APC (95% CI)** | **Sensitivity segment** | **Sensitivity APC (95% CI)** | **Notes** |
| --- | --- | --- | --- | --- | --- |
| **Overall** | 2015–2023 | +1.25%* (0.38, 2.13) | 2015–2019 | +0.29% (−2.11, 2.75) | p=0.80; no longer significant |
| **Female** | 2016–2023 | +1.98%* (0.94, 3.03) | 2016–2019 | +2.71% (−0.72, 6.27) | p=0.11; point estimate preserved, power lost |
| **Male** | 2014–2023 | +0.58% (−0.71, 1.90) | 2014–2019 | −0.52% (−3.92, 3.00) | p=0.75; stable in both windows |
| **45–64 years** | 2013–2023 | +3.24%* (1.39, 5.13) | 2012–2019 | +0.20% (−2.70, 3.19) | p=0.89; no longer significant |
| **65–85+ years** | 2015–2023 | +0.47% (−0.63, 1.58) | 2015–2019 | −0.29% (−2.73, 2.10) | p=0.80; stable in both windows |
| **NH Black/African American** | 2016–2023 | +2.87%* (0.26, 5.56) | 2016–2019 | +1.93% (−6.14, 10.68) | p=0.63; no longer significant |
| **NH White** | 2015–2023 | +1.38%* (0.31, 2.46) | 2015–2019 | +0.40% (−2.77, 3.67) | p=0.79; no longer significant |
| **NH Asian/Pacific Islander** | 2014–2023 | −0.74% (−4.07, 2.71) | 1999–2019 (0 joinpoints) | −7.15%* (−8.18, −6.10) | No reversal detected in either window |
| **Hispanic/Latino** | 2015–2023 | +2.96% (−0.19, 6.21) | 2015–2019 | +1.51% (−7.02, 10.83) | p=0.72; not significant in either window |
| **Census: Northeast** | 2014–2023 | +0.26% (−1.68, 2.23) | 2015–2019 | +3.12% (−1.78, 8.26) | p=0.20; not significant in either window |
| **Census: Midwest** | 2015–2023 | +1.47% (−0.46, 3.44) | 2015–2019 | +0.03% (−5.71, 6.12) | p=0.99; not significant in either window |
| **Census: South** | 2017–2023 | +3.16%* (0.51, 5.87) | 2009–2019 | −4.81%* (−6.02, −3.58) | No reversal joinpoint detected pre-2020 |
| **Census: West** | 2014–2023 | +1.09% (−0.51, 2.71) | 2010–2019 | −2.86%* (−4.49, −1.20) | No reversal joinpoint detected pre-2020 |
| **Metropolitan** | 2015–2020 | −0.51% (−2.36, 1.37) | 2015–2019 | −0.16% (−2.82, 2.58) | p=0.90; negligible change |
| **Non-metropolitan** | 2015–2020 | +1.92% (−1.55, 5.51) | 2015–2019 | +1.69% (−3.94, 7.33) | p=0.50; negligible change |

* Statistically significant at α=0.05 in that analysis. CI = 95% confidence interval. "No reversal joinpoint detected" indicates the best-fit Joinpoint model for 1999–2019 did not identify a late-period upward segment; the final listed segment continued to decline.

**Note:** P-values represent the statistical significance tests for the corresponding Annual Percentage Change (APC) estimates from the Joinpoint regression models; p<0.05 was considered statistically significant.
